# Aptamer-conjugated gold nanoparticles and their diagnostic and therapeutic roles in cancer

**DOI:** 10.3389/fbioe.2023.1118546

**Published:** 2023-01-19

**Authors:** Guozhen Deng, He Zha, Hongzhi Luo, Yi Zhou

**Affiliations:** ^1^ Department of Laboratory Medicine, The First People’s Hospital of Zunyi (The Third Affiliated Hospital of Zunyi Medical University), Zunyi, Guizhou, China; ^2^ Department of Orthopaedics, Jian Yang Hospital of Traditional Chinese Medicine, JianYang, Sichuan, China

**Keywords:** aptamer, gold nanoparticles, cancer diagnosis, cancer targeted, therapy

## Abstract

The burden of incidence rate and mortality of cancer is increasing rapidly, and the development of precise intervention measures for cancer detection and treatment will help reduce the burden and pain of cancer. At present, the sensitivity and specificity of tumor markers such as CEA and CA-125 used clinically are low, while PET, SPECT, and other imaging diagnoses with high sensitivity possess shortcomings, including long durations to obtain formal reports and the inability to identify the molecular pathological type of cancer. Cancer surgery is limited by stage and easy to recur. Radiotherapy and chemotherapy often cause damage to normal tissues, leading to evident side effects. Aptamers can selectively and exclusively bind to biomarkers and have, therefore, gained attention as ligands to be targeted for cancer detection and treatment. Gold nanoparticles (AuNPs) are considered as promising nano carriers for cancer diagnosis and treatment due to their strong light scattering characteristics, effective biocompatibility, and easy surface modification with targeted agents. The aptamer-gold nanoparticles targeting delivery system developed herein can combine the advantages of aptamers and gold nanoparticles, and shows excellent targeting, high specificity, low immunogenicity, minor side effects, etc., which builds a bridge for cancer markers to be used in early and efficient diagnosis and precise treatment. In this review, we summarize the latest progress in the application of aptamer-modified gold nanoparticles in cancer targeted diagnosis and delivery of therapeutic agents to cancer cells and emphasize the prospects and challenges of transforming these studies into clinical applications.

## 1 Introduction

Cancer is one of the leading causes of death worldwide, and it is estimated that nearly one-sixth of the global population (approximately 10 million) have died as a consequence of the disease until 2020. The most common cancer types include breast cancer, lung cancer, colon cancer, rectal cancer, and prostate cancer (de Martel et al., 2020). It is essential to perform an accurate diagnosis of cancer and take appropriate and effective treatment measures, which can greatly improve the prognosis and survival rate of cancer patients (Sung et al., 2021). For several years, researchers have devoted themselves to finding additional sensitive and specific biomarkers related to cancer. However, there is still a lack of effective methods to detect cancer biomarkers. Meanwhile, to improve the effectiveness and precision of medical treatments, new therapies combined with cancer biomarkers must be developed (Zou and Wang, 2020). Therefore, it is crucial to explore the usage of such markers to diagnose diseases more accurately and use them for targeted cancer treatment.

An aptamer is an unnatural oligonucleotide consisting of 25–90 nucleotides (usually RNA or DNA), which can be folded into a complex three-dimensional structure through intramolecular interactions (Ellington and Szostak, 1990). Adapters can selectively and exclusively identify various types of targets, such as proteins (Chen et al., 2019), drugs (Parker et al., 2019), heavy metal ions (Guo et al., 2021), toxins (di Leandro et al., 2021), and cells (Kordasht and Hasanzadeh, 2020). For a long time, immunological and genomic methods have dominated the detection market for tumour markers (Gao et al., 2019). Aptamers have recently enticed increasing attention by reason of their unique properties. An aptamer has a binding affinity to the target, however, it is different from the immunological antibody that recognizes the target by binding antigen and antibody. Aptamers act specifically with homologous molecular targets based on its specific three-dimensional structure (Keefe et al., 2010). Furthermore, the volume of the adapter allows itself to be densely arranged on the sensor surface to enhance the sensitivity of the biosensor (Jung et al., 2020). Ligands can not only be used as independent therapeutic agents, but also as carriers for targeted drug delivery. The specific recognition, low immunogenicity, and low cytotoxicity of aptamers can increase the drug concentration in tumour tissues and reduce side effects (Wang et al., 2022). Compared to the conventional treatment scheme for tumours, adapters can avoid the high recurrence rate of surgical resection, the systemic side effects caused by drug chemotherapy, and the cumulative radiation of radiotherapy. Aptamers also have the characteristics of simple preparation process, easy mass production, and good organizational penetration (Chen et al., 2017; Xu et al., 2021). Such numerous advantages of aptamers indicate that they possess excellent applicability for the diagnosis and treatment of tumours.

In addition, with the rise of tumour nanotechnology, the efficiency of tumour diagnosis and treatment has improved. The development of new anti-tumour drugs based on gold nanoparticle (AuNPs) has become a new hot research field for tumour nanotechnology. AuNPs can not only perform functions independently at the cellular or molecular level, but also possess the ability to integrate multiple functions. Researchers used the characteristics of AuNPs to construct the aptamer-conjugated AuNPs for tumour diagnosis and treatment. An immunosensor can be made more sensitive by amplification of the signals using AuNPs after the adapter conjugates with them during the detection of cancer-specific markers (Qian et al., 2020). Simultaneously, AuNPs has unique surface plasmon resonance (SPR) and adjustable surface functionality (fluorescent dyes, redox markers, and other chemical modifications can be easily carried out on AuNPs), because of which the detection results can be visualized using not only immunosensors but also computed tomography images, microscopy, and additional techniques (Jimenez et al., 2019; Liao et al., 2019; Chakraborty et al., 2021). For cancer treatment, the active targeting of aptamers can more accurately select target cells to assist in the controlled and continuous transfer of therapeutic molecules (drugs (Go et al., 2021) and peptides (Kim et al., 2015)) using AuNPs and improve the uptake of therapeutic molecules by the cells. It is worth noting that the unique photothermal conversion characteristics of AuNPs makes it a suitable photosensitizer for the photothermal treatment of colorectal cancer and additional tumours(Chugh et al., 2018; Wang et al., 2021). AuNPs is excited at a specific wavelength, and the vibration energy is emitted in the form of heat to destroy target cells without damaging normal tissues (Gupta and Malviya, 2021). Therefore, the photothermal conversion characteristics of AuNPs can not only make the treatment of tumour using the aptamer no longer limited to targeted drug delivery, but can also kill tumour tissues through photothermal therapy. In other words, the combination of the two features can overcome the limitations of traditional tumour diagnosis and treatment.

The progress of precision medicine has provided a breakthrough for the detection of cancer targeting molecules and personalized precision therapy. In 2015, President Obama announced in his State of the Union address that the United States had launched the "Precision Medical Initiative", which promoted global attention and application research on the concept of precision medicine. Similarly, the "China Individualized Medicine Precision Medical Science Industry Alliance" was officially established in Shanghai, moving towards the integration of industry, education, and research in the field of precision medicine. A significant amount of money and energy have been invested in high-throughput genome sequencing in the early work of precision medicine (Nakagawa and Fujita, 2018). The molecular markers discovered using sequencing technology provide a basis for precision medicine. To maximize the effectiveness of precision medicine, precise diagnosis and treatment are required. In certain basic experiments, few biomarkers that are accurate, reliable, and related to the prognosis of the disease are used as molecular targets for cancer diagnosis or treatment using adapters and AuNPs. For example, WuY et al. realized the recognition of ER2 and HER2, two cell classification markers for breast cancer, using double aptamer AuNPs (Wu et al., 2022). Ruttala HB et al. achieved combined killing of tumour cells such as MCF-7, MDA-MB-231, and DU145 by drugs and photothermal therapy through AS1411Apt AuNP-loaded anisodamine combined with infrared thermotherapy (Ruttala et al., 2020). *In vivo* or *in vitro* experiments have fully verified the potential diagnostic function and therapeutic potential of the ligand combined with AuNPs.

**FIGURE 1:**
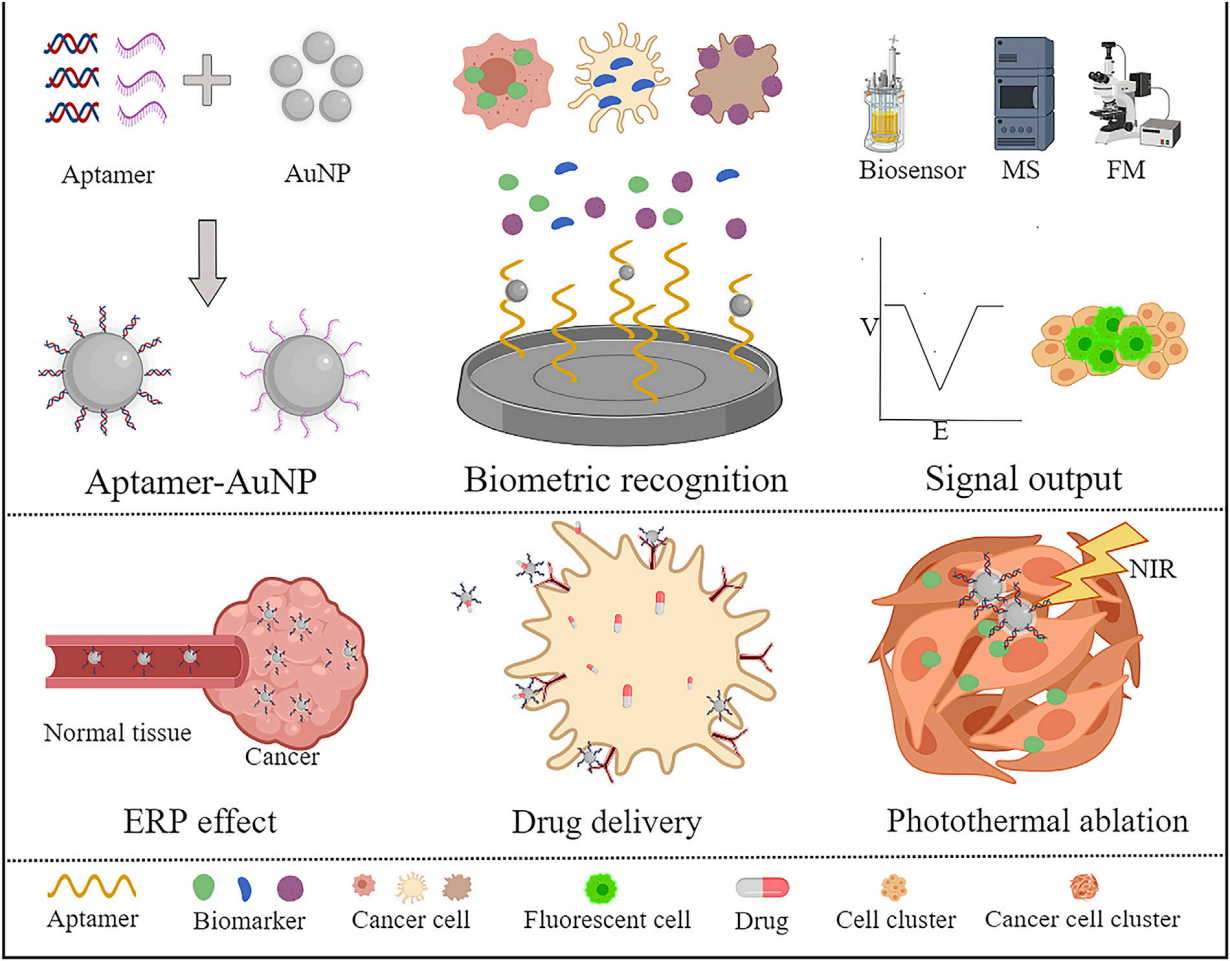
Application of aptamers combined with AuNPs for the diagnosis and treatment of tumours. A variety of special tumour markers have been developed based on AuNP binding with aptamers. The detection results can be visualized by combining biosensors, computed tomography, fluorescence microscopes, etc. The researchers also targeted tumour therapy with drug carriers and photothermal therapy through AuNP junction suitable ligands.

The purpose of the review is to investigate the progress in the application of aptamer-modified AuNPs in cancer marker detection and targeted therapy on the basis of the characteristics and advantages of aptamers and AuNPs (Figure 1). Finally, we analyse the prospects and challenges associated with the advancement of AuNPs from the current basic research to clinical experiments, which may help achieve personalized precision medicines.

## 2 Superiority of aptamers as targeting ligands

Aptamers are short oligonucleotide DNA or RNA oligonucleotides formed by base pairing into three-dimensional structures. Aptamer comes from Latin words "aptus" or "aptare" (Darmostuk et al., 2015; Ellington and Szostak, 1990; Sefah et al., 2010), which means a nucleotide polymer that is suitable for and can combine with the target with high specificity and affinity. For a long time, antibodies have been widely used in medical practice. Recently, the unique advantages of aptamers have challenged the role of antibodies in *in vitro* diagnosis and drug therapy. Nucleic acid aptamers were screened by exponential enrichment ligand phylogenetic technology (SELEX). The obtained aptamer was sequenced, characterized and identified. The qualified aptamer can be used as an excellent target molecule (Kong and Byun, 2015; Zhang et al., 2019). Compared to antibodies, the production time of aptamers is only several hours, the production cost is low, and the difference between batches is small. A stable three-dimensional structure is the basis for an aptamer to perform the target recognition function (Hermann and Patel, 2000), which makes it different from antibodies that depend on antigen-antibody binding. Adapters can recognize the targets of proteins (Farrow et al., 2022), drugs(Tang et al., 2020), metal ions(Guo et al., 2021), toxins(di Leandro et al., 2021), cells(Kordasht and Hasanzadeh, 2020) and other types of substances. In addition, aptamers also have the advantages of easy modification or coupling of functional groups, strong tissue penetration, suitable thermal/chemical stability, and low immunogenicity. Therefore, aptamers having such characteristics have been used in molecular recognition elements to detect several diseases, such as using G-quadruplex to form DNA aptamers to detect SARS CoV2(Mandal et al., 2021). The aptamers combined with AuNPs have realized the diagnosis of breast cancer with high HER2 expression(Zhu et al., 2013). As early as 2004, Macugen ^®^ (pegatanib) has been approved by the US Food and Drug Administration as the first adapter drug. Presently, various fitness-based diagnoses are also in full swing in preclinical research and clinical trials. Adapters combined with AuNPs can be used for targeted drug therapy and photothermal therapy of cervical cancer(Li M et al., 2021), lung cancer(Yang Y et al., 2021), and colorectal cancer(Go et al., 2021) by carrying drugs or using the optical properties of AuNPs, thereby reducing the side effects on normal tissues. In conclusion, aptamers have a wide range of physical and biochemical properties that demonstrate their potential as both diagnostic tools and therapeutic agents, and can be considered as suitable application prospects in clinical disease diagnosis and treatment.

## 3 Unique characterization of AuNPs and their applicability in diagnosis and treatment

Although NPs are related to modern science and technology, AuNPs has been used since Paracelsus (i.e., the founder of modern chemistry in Europe in the 16th century) prepared "drinking gold" to treat mental diseases. AuNPs refer to tiny particles of gold with a diameter between 1 and 100 nm. Among various organic and inorganic nanoparticles, the unique physical, Chemical, and biological characteristics of AuNPs provide several possibilities for disease diagnosis, drug delivery, and photothermal treatment. The optical resonance and SPR of AuNPs endow the gold particles with the ability to absorb and scatter visible light(Kumar et al., 2007). When AuNPs are focused on a target location, The target object can be visualized through phase contrast optical microscope, dark field microscope, photothermal imaging, and photoacoustic imaging. The strong optical scattering characteristics and relative histocompatibility of AuNPs are also used to detect tumour markers. In surface-enhanced Raman spectroscopy (SERS), AuNPs can amplify the Raman signal of the detection molecules several times; Thus, AuNPs are the most popular nanostructures used as sensors presently(Das et al., 2021). Under near infrared (NIR) irradiation, AuNPs show extremely strong light-heat conversion efficiency, which increases the local temperature of the tumour site, leading to apoptosis of the tumour cells that are extremely sensitive to heat(Singh et al., 2018). The enhanced permeability and retention (ERP) effect of the nanoparticles increases its targeting ability by 10–100 times compared to that of small molecule drugs(Kalyane et al., 2019). Furthermore, AuNPs also have a large body surface area, which can be used to couple biological recognition fragments (such as nucleic acid aptamers(Mirau et al., 2018), proteins(Lira et al., 2018), antibody fragments(Veigas et al., 2019), peptides(Garaiova et al., 2021), etc.,) to further enhance the targeting specificity or therapeutic diversity. Currently, numerous experimental results show aptamers combined with AuNPs can be applied for the diagnosis and treatment of tumours, providing a new direction for tumour-targeted therapy.

## 4 Aptamer-conjugated AuNPs in cancer diagnostics

There are superior aptamer-based sensors (aptasensors) to antibody-based biosensors. Specifically, aptamers are suitable for tumour diagnosis due to their stability, high affinity for the target, high specificity, and low production costs (Sahin et al., 2020; Mo et al., 2022). An aptamer is used as a probe to construct a nano-sensor with AuNPs that can amplify various signals, such as colorimetric (Sharifi et al., 2020), fluorescent (Cheng et al., 2018), Electrochemical (Wei et al., 2018), or optical signals (Wang et al., 2020). Furthermore, AuNPs are highly X-ray absorbent and non-toxic, making them excellent contrast agents. AuNPs coupled with aptamers are highly specific, sensitive, and safe for tumour diagnosis. Therefore, they have an important application prospect for the diagnosis of tumours (Table 1).

**TABLE 1:** Apatamer-AuNP based detecting platforms for cancer diagnose.

Aptamer-AuNP	Detecting platform	Target molecule	Target disease	Target cell line	Aptamer sequence	Ref
A 3′-thiolated RNA	Visualized by the bare eye and an optical microscope, quantitatively analyzed using stripping voltammetry	HER2	Breast cancer	HER2-positive breast cancer cells	SE15-8, 3′-AAA​AGT​TGT​GAG​GGG​AGG​GAT AGG​GTA​GGG​CAC​GAC​TAG​TCA​AGA​AAA​TG-5′	Zhu et al. (2013)
Aptamer-AuNP						
PSMA Aptamer-GNP	SWV	PSA	Prostatic cancer	LNCaP cell	5′-GGG​AGG​ACG​AUG​CGG​ACC​GAA​AAA​G ACC​UGA​CUU​CUA​UAC​UAA​GUC​UAC​GUC​CCA​GAC​GAC​UCG​CCC​GAC​GAC​GAC​GAC​GAC​GAC​GAC​GA-3′	Li M et al. (2021)
Aptamer (labeled with MB)-AuNP-signal probe	CT	PSA	Prostatic cancer	LNCaP cell and PC3 cell	Aptamer: 5′-SH-(CH2)6TTTTTAATTAAAGC TCG​CCA​TCA​AAT​AGC​TTT-3′	Kim et al. (2010)
Dual-aptamers (_t_AH and _f_AE)-GNP	FRET	ER and HER2	Breast cancer	MCF-7 cell, SK-RB-3 cell, MDA-MB-231 cells	_t_AH 5′-TAMRAGCAGCGGTGTGGGGGCAG CGG​TGT​GGG​GGC​AGC​GGT​GTG​GGG-3′, _f_AE 5′-FAM-CCCGGCATGGTTGCGGAGCA GGA​GTA​TAA​CAC​TAC​CAT​TG-3′	Wu et al. (2022)
AS1411 aptamers-GNP	X-ray/CT imaging	Nucleolin and MMP-14	Cancer with high expression of nucleolin and MMP-14	MDA-MB 231 cell	AS1411 (Integrated DNA Technologies; Coralville, IA)	Kang and Nguyen (2017)
AptMUC1–AuNPs	GO–LDI-MS	MUC1	Breast cancer	MCF-10A cell and MCF-7 cells	5′-HS-TTTTTTTTTTTTTTTGCAGTTGATCC TTTGGATACCCTG G-3′	Huang et al. (2015)

AuNP, gold nanoparticles; HER2, human epidermal growth factor receptor 2; SWV, square wave voltammetry; PSMA, prostate specific membrane antigen; PSA, prostate specific antigen; FRET, förster resonance energy transfer; CT, computed tomography; ER, estrogen receptor; MMP-14, matrix metallo-proteinase 14; MUC1, mucin1; LDI-MS, laser desorption/ionization mass spectrometry.

### 4.1 Hydrazine—AuNP—aptamer bioconjugate

**FIGURE 2:**
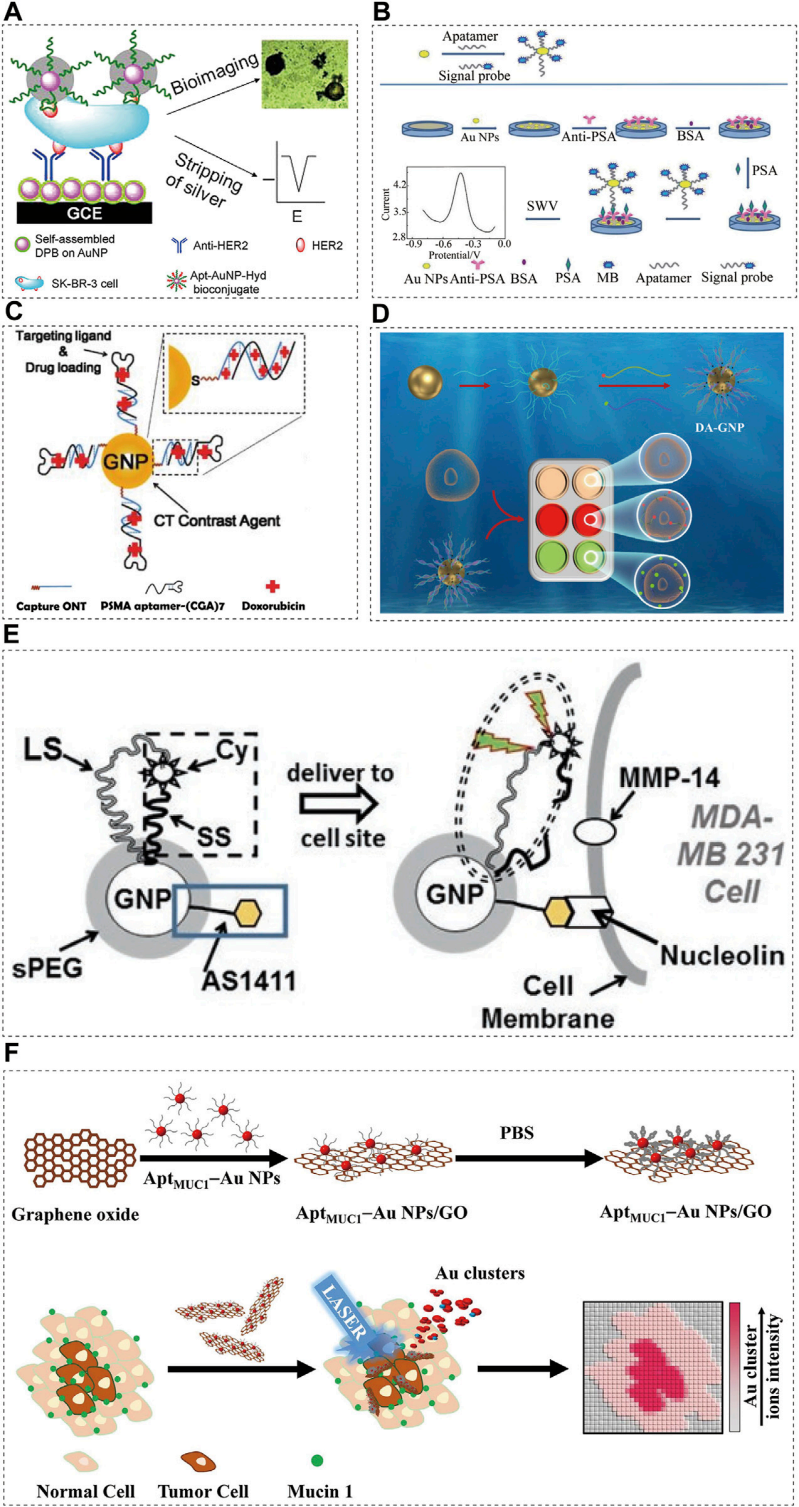
Aptamer-conjugated AuNPs in cancer diagnostics. Schematic illustrations showing: **(A)** Schematic representation of immunosensors for the detection of HER2 protein and HER2-overexpresing SK-BR-3 breast cancer cells, reprinted with permission from (Zhu et al., 2013), Copyright 2013, American Chemical Society; **(B)** Assembly and detection process of the immunosensor, reprinted with permission from (Li M et al., 2021), Copyright 2022, American Chemical Society; **(C)** Schematic illustration of the method for preparing Dox-loaded aptamer-conjugated AuNPs (Kim et al., 2010), Copyright 2010, ACS Publications; **(D)** Illustration of classification of breast cancer by DA-GNP, aptamer-conjugated AuNPs (Wu et al., 2022), Copyright 2022, Elsevier; **(E)** Schematic diagram of AuNP-based optical/CT contrast agent for MDA-MB 231, aptamer-conjugated AuNPs (Kang and Nguyen, 2017), Copyright 2017, Spinger eBook; **(F)** Schematic representation of the preparation of MUC1-binding aptamer–modified AuNPs (AptMUC1–Au NPs) and their conjugation to graphene oxide (AptMUC1–Au NPs/GO) for tumour tissue imaging by monitoring Au cluster ions when coupled with laser desorption/ionization mass spectrometry, aptamer-conjugated AuNPs (Huang et al., 2015), Copyright 2015, Spinger Nature.

In 10–25% of breast cancer cases, a key prognostic marker and effective therapeutic target for breast cancer is the human epidermal growth factor receptor 2 (HER2) (Slamon et al., 1987; Owens et al., 2004). A significant amount of overwhelming evidence shows that patients with HER2 positive breast cancer have a worse prognosis than those with HER2 negative breast cancer, and special form of treatment is required for HER2 positive patients (Hung et al., 1986; Telli et al., 2007). For this reason, HER2 overexpression is crucial for breast cancer diagnosis and treatment. In recent years, nanomaterials (AuNPs or graphene) have been used to detect DNA and protein by combined silver deposition (Taton et al., 2000; Chumbimuni-Torres et al., 2006). A reducing agent (such as hydroquinone) is used to chemically reduce silver ions on the sensor surface during these determinations, resulting in non-specific silver deposition on the sensor surface, which makes the detection unrepeatable. In order to overcome the problem of silver deposition on sensors, a new method of silver reduction without external reducing agents was proposed by Zhu et al. (2013). An electrochemical immunosensor combined with AuNPs containing hydrazine and an aptamer detected HER2-overexpressing breast cancer cells. The sensor probe is prepared by covalently immobilizing the anti HER2 antibody on the nanocomposite layer. The nanocomposite layer is composed of AuNP assembled with 2,5-bis (2-thienyl) - 1H-pyrrol-1 - (p-benzoic acid) (DPB). By directly attaching the hydrazine reductant to the AuNP, non-specific silver deposition can be avoided on the sensor surface and signal enhancement is possible by selectively reducing the silver ions (Figure 2A). Using a light microscope or naked eye, silver-stained target cells can be observed easily. Thus, HER2 positive and negative breast cancer cells can be distinguished. Combined with stripping voltammetry, HER2 expressed in the cells can be quantitatively analysed. SK-BR-3 breast cancer cells can also be detected using this method in serum samples from humans, providing a simpler option for cancer cell detection.

### 4.2 Drug-loaded prostate specific membrane antigen (PSMA) aptamer AuNP bioconjugate

In a study on the targeted diagnosis of prostate cancer, Popovtzer et al. (Popovtzer et al., 2008) utilised the characteristics of AuNPs as an imaging agent for the visualization of magnetic resonance imaging (MRI) or optical imaging. It has been reported that PSMA-specific aptamers bound to multifunctional gold nanoprobes (GNPs) can be used as anti-cancer drug delivery carriers and CT nano contrast agents. A contrast agent based on GNPs could overcome the limitations of traditional contrast agents based on iodine, including lack of targeted molecular imaging and nephrotoxicity (Kim et al., 2007). The obtained PSMA aptamer-conjugated GNP showed that the CT intensity of the targeting LNCaP cells was more than four times higher than that of the non-targeting PC3 cells. Furthermore, PSMA-specific aptamers form 1:1 complexes with doxorubicin (DOX) *via* intercalation, delivering the anti-cancer medication to the prostate cancer cells (Figure 2C). After loading adriamycin, the effect of the PSMA-aptamer-bound GNPs on the targeting LNCaP cells was significantly higher than that on the non-targeting PC3 cells (Kim et al., 2010).

### 4.3 Prostate specific antigen (PSA) aptamer-gold nanoparticle-signal probe bioconjugates

PSA is a serum biomarker associated with prostate cancer (PCa). The PSA level of normal healthy subjects is lower than 4.0 ng/mL. In the grey area (4–10 ng/mL), the current prostate cancer detection method has reduced specificity when PSA levels are in this range (Healy et al., 2007). The development of a sensitive method for the detection of low PSA abundance will contribute to the diagnosis of early prostate cancer. Presently, aptamer-based PSA immunoassays have been reported, based on electrochemistry (Hu et al., 2020; Zheng et al., 2021), fluorescence (Pei et al., 2015), and chemiluminescence (Shi et al., 2016). Due to their simplicity, sensitivity, and high selectivity, electrochemical methods have gained increasing attention. In one study (Li M et al., 2021), the detection of the prostate cancer biomarker PSA with an electrochemical immunosensor was recently reported as a simple and sensitive method. In the design scheme, AuNPs were used as the carrier to load the aptamer, and methylene blue (MB, a redox signal probe) was used to label the aptamer to synthesize AASp complex. By immobilizing the antibody on the AuNP-modified electrode surface to capture the PSA antigen, the AASp complex recognized the PSA antigen to form a sandwich structure (Figure 2B). The signal at approximately 0.45 V was detected using square wave voltammetry and a well-shaped peak corresponding to MB oxidation was obtained. PSA concentration was related to peak intensity. The linear response range was widened (0.001–75.0 ng/ml) and the detection limit was reduced to 3.0 pg/mL due to an AASp conjugate amplification of the detected signal.

### 4.4 Dual-aptamers functionalized gold nanoprobe (DA-AuNP)

In most studies on cancer diagnosis with aptamer sensors, a single cancer specific aptamer probe for a single cancer cell line is used (Razmi et al., 2018; Samanta et al., 2020; Li L et al., 2021). Because most cancers are highly heterogeneous in their development, single aptamer cancer probes lack the sensitivity and specificity needed for detection, and a single biomarker cannot represent the tumour pathogenesis and stage caused by multiple genetic changes (Colomer et al., 2018). Dual-aptamer functionalized nanoprobes can simultaneously target different epitopes expressed in multiple types of breast cancer because of the multivalent effect of nanomaterials, which can improve the accuracy of detection (Li et al., 2011). In another study (Wu et al., 2022) on the diagnosis of breast cancer, a double-aptamer-functionalized gold nanoprobe (DA-GNP) based on Förster resonance energy transfer (FRET) was designed for the classification of breast cancer (Figure 2D). Fluorescent labelled ER and HER2 specific aptamers were hybridized with the corresponding probe oligonucleotides to form double stranded bodies. The specific combination of the aptamer with its specific breast cancer subtype biomarker induces the targeted aptamer to leave the GNP surface, resulting in fluorescence recovery. The unique FRET based aptamer sensor allows quantitative classification of different breast cancer subtypes.

### 4.5 AS1411 aptamer-AuNPs

Multiple functions can be integrated into one AuNP and remain in the body for a longer period of time. The effective light scattering characteristics and relative biocompatibility of AuNPs make them a natural X-ray/CT contrast agent (Aryal et al., 2017). Using such beneficial aspects of nanotechnology, multifunctional contrast agents based on AuNPs can be developed for selected types of cancer. Usually, nucleolin is found in the nucleus, however, it is overexpressed on the surface of the cell membrane in various cancers. It is known that the DNA aptamer AS1411 is an excellent target for nucleolin, which is overexpressed on the surface of the cancer cells (Yazdian-Robati et al., 2020). MMP-14 is an enzyme expressed on the cell membrane that promotes cancer metastasis and angiogenesis (Winer et al., 2018). The unique ability of AuNPs to regulate fluorescence was used to trigger fluorescence in AS1411 at an enhanced level, making it a cancer targeting molecule (Kang and Wang, 2014; Kang and Nguyen, 2017). The short spacer (SS) is short in design and closely combines Cyate with AuNP; the long spacer (LS) allows Cyate to be placed at a certain distance from AuNP (Figure 2E). This synthetic reagent usually emits little fluorescence until it binds to the cancer cells through AS1411 and encounters MMP-14. Subsequently, the SS is cut and Cypat is released from the SS, which causes the distance between Cypat and AuNP to become the whole LS length, thereby generating enhanced fluorescence. Through fluorophore, the contrast agent is sensitive to optical imaging, and it provides suitable resolution for X-rays and CTs (Figure 2E) (Kang and Nguyen, 2017).

### 4.6 AptMUC1-conjugated gold nanoparticles immobilized

In most adenocarcinomas, mucin 1 (MUC1) is overexpressed, making it an attractive target for cancer biomarkers (Nath and Mukherjee, 2014). Nanoparticle-based imaging systems were combined with laser desorption/ionization mass spectrometry (LDI-MS) in a study (Huang et al., 2015), employing the MUC1 junction suitable ligand (AptMUC1) as the targeting agent (Figure 2F). The MS-based tissue imaging method can be performed without the use of fluorescent probes or radioactive labels, which makes it an attractive option (Norris and Caprioli, 2013; Weaver and Hummon, 2013). Moreover, MS imaging (MSI) is a highly sensitive, fast, and multiplex technology that can identify multiple complex biomolecules simultaneously. The researchers (Huang et al., 2015) used LDI-MS analysis to prove that the nanocomposite AptMUC1–AuNPs/GO selectively binds to MCF-7 cells. When Apt-AuNP/GO combine with the tumour cells, they can present highly amplified Au cluster ion signals which can be monitored using LDI-MS.

## 5 Aptamer-conjugated AuNPs for cancer therapeutics

### 5.1 Drug delivery

For the treatment of malignant tumours, several drugs lack the specificity of the targeting tumour cells, causing damage to the normal cells. As a target recognition ligand, an aptamer can distinguish between normal and cancer cells. By conjugating aptamers with NPs, a drug targeting delivery system can be created (Table 2), which can selectively deliver cytotoxic drugs to tumour cells and reduce the toxic side effects on normal cells. The low toxicity, non-immunogenicity, biocompatibility, and adjustable surface functionality of AuNPs make them attractive carriers for drug delivery (Li et al., 2019; Sharifi et al., 2019). In terms of engineering and application, ligand-bound AuNPs provide a powerful platform for target recognition and treatment, and hold potential in tumour therapy. In addition, aptamer-AuNP targeted drug delivery systems possess the advantages of high stability, high drug loading, and improved drug bioavailability (Pelaz et al., 2012).

**TABLE 2:** Apatamer-AuNP based detecting platforms for cancer therapeutic.

Treatment methods	Aptamer-AuNP	Target molecule	Target disease	Therapeutic agents/Physical exposure	Aptamer sequence	Ref
Drug dilivery	AS1411 aptamers	Nucleolin	Cervical cancer	C8 or IQ; Dox	AS1411 was purchased from Eurogentec(Liege, Belgium)	Lopes-Nunes et al. (2021)
				TMPyP4 and Dox	strand1:5′-thiol-TTTTTTTTTTTCGATCGTCGATCGTCGATCG)-3′,strand2:5′-GGTGGTGGTGGTTGTGGTGGTGGGGTTTTTTCGATCGACGATCGACGATCGA-3′)	Shiao et al. (2014)
			Lung cancer	Dox and siVEGF	—	Yang Y et al. (2021)
			Gastric cancer	Morin hydrate	5′-HS-T-(C6-S-S-C6)-TTGGTGGTGGTGGTTGTGGTGGTGGTGG-3′	Ding et al. (2020)
	PrP^C^ aptamer	PrP^C^	Colorectal cancer	Dox	5′-HS-C6-AAAAAAAAAA-TCG-TCG-TCG-TCG-TCGTCG-TCG-CGGTGGGGCAATTTCTCCTACTGT-3′, underlined sequence is for the PrPC	Go et al. (2021)
	AuNP–His/GST-Apt	DDR2	Cervical cancer	BIM or EGF	anti-His aptamers: (5′-GCT​ATG​GGT​GGT​CTG​GTT​GGG​ATT​GGC​CCC​GGG AGCTGGC-A10-Thiol-3′) anti-GST aptamers: (5′-CTGCCCCGCTATAGA AC ACCCGTTGGGCAAATGTGTTCGA-A10-Thiol-3′)	Ryou et al. (2014)
			Lung cancer	Peptides containing DDR2 or labeled transmembrane proximal 1/2	—	Kim et al. (2015)
Photothermal therapy	Apt-AuNP-GO	MUC1	Breast cancer	NIR	—	Yang et al. (2015)
			Lung cancer	DOX/NIR	S2.2 aptamer: 5′-HS-GCAGTTGATCCTTTGGATACCCTG- FAM -3′	Wang et al. (2015)
	AS1411-GNP	Nucleolin	MCF-7,MDA-MB-231, DU145 cell	LND/Laser thermotherapy	50-amino-C6 linker; NH2-5′-(GGTGGTGGTGGTTGTGGTGGTGGTGG) -3′	Ruttala et al. (2020)

AuNP, gold nanoparticles; C8, acridine orange derivative; IQ, imiquimod; Dox, doxorubicin; TMPyP4, 5,10,15,20-tetrakis(1-methylpyridinium-4-yl) porphyrin; VEGF, the vascular endothelial growth factor; PrP^C^, the cellular prion protein; DDR2, discoidin domain receptor 2; EGF, epidermal growth factor; MUC1, mucin1; NIR, near-infrared; GNP, gold nanoparticles; LND, lonidamine.

#### 5.1.1 AS1411 aptamer conjugated-gold nanoparticle

AS1411 aptamers are guanine-rich DNA nucleic acid aptamers composed of 26 nucleotides. They are non-SELEX screened nucleic acid aptamers with a G-quadruplex structure. The AS1411 aptamer has good thermal stability and no immunogenicity, can resist nuclease in serum, and shows strong stability (Shigdar et al., 2021). Presently, targeted drug delivery systems consisting of the AS1411 aptamer as a targeting ligand, AuNP as a carrier, and a drug have been studied for a variety of treatments.

##### 5.1.1.1 Cervical cancer

**FIGURE 3:**
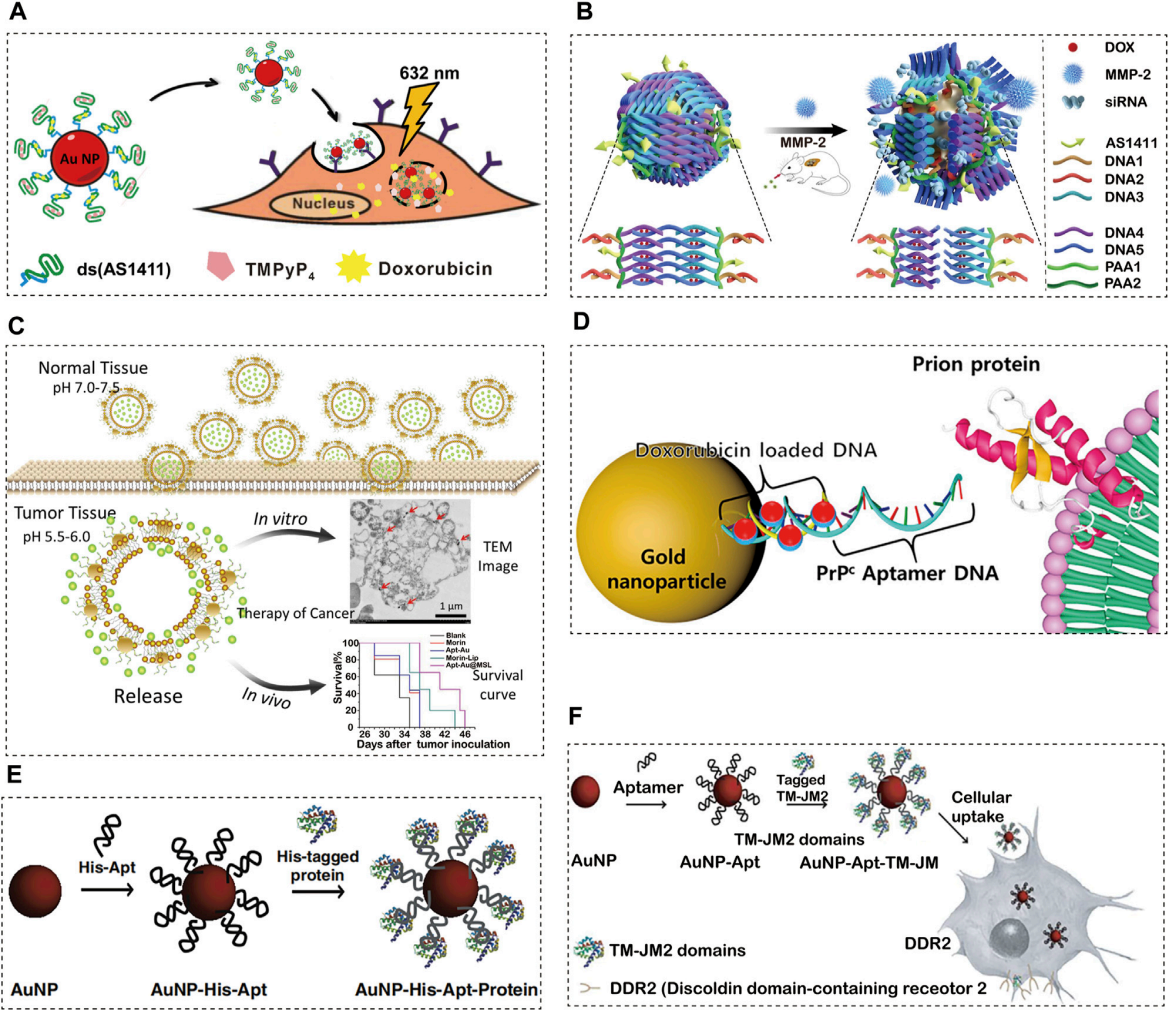
Aptamer-conjugated AuNPs for cancer therapeutics. **(A)** Schematic Illustration of the Co-Drug-Loaded Aptamer-Functionalized Delivery Platform on the Basis of Gold Nanoparticles, reprinted with permission from(Lopes-Nunes et al., 2021), Copyright 2014, American Cemical Society; **(B)** The schematic illustration of the construction of gold nanocages and the tumour-induced gene- and DOX-releasing mechanism, reprinted with permission from(Yang Y et al., 2021), Copyright 2021, Spinger Nature; **(C)** Proposed schematic diagram of designed Apt-Au@MSL containing Morin for drug delivery to cancer cells with tumour-targeted property, reprinted with permission from(Ding et al., 2020), Copyright 2020, Nanoscale Research Letters; **(D)** Synthesis and characterization of PrPC-Apt-functionalized doxorubicin-oligomer-AuNPs (PrPC-Apt DOA), reprinted with permission from(Go et al., 2021), Copyright 2021, Molecular Diversity Preservation International; **(E)** Proposed schematic diagram of our designed Apt-Au@MSL containing Morin for drug delivery to cancer cells with tumour-targeted property, reprinted with permission from(Ryou et al., 2014), Copyright 2014, Elsevier; **(F)** Schematic diagram of the generation of TM-JM1/2 peptides containing the aptamer-conjugated AuNP delivery system, reprinted with permission from(Kim et al., 2015), Copyright 2015, Elsevier.

In cervical cancer, one study (Lopes-Nunes et al., 2021) covalently bound the AS1411 aptamer with AuNPs as the carrier of acridine orange derivative (C8) or imiquimod (IQ) (Figure 3A). Upon final synthesis, the nanoparticles possess characteristics that are suitable for drug applications, such as small size, negative charge, and effective release of drugs. The nanoparticles were then added to a polyethylene glycol gel preparation, and the gel showed adequate tissue retention characteristics in a Franz cell study of porcine vaginal epithelium. AS1411 AuNPs have demonstrated efficacy as drug carriers for cervical cancer treatment based on these findings. They can overcome the lack of selectivity of C8 potential anti-cancer ligand, and can improve the anti-cancer effect of the commercially available drug (imiquimod). In another study (Shiao et al., 2014), the photosensitizer 5,10,15,20-tetra (1-methylpyridin-4-yl) porphyrin (TMPyP4) and chemotherapy drug DOX were physically attached to AS1411-conjugated AuNPs and delivered to HeLa and DOX resistant MCF-7R cell lines. The cells were damaged by the reactive oxygen species (ROS) produced by the TMPyP4 molecules when they were exposed to light at 632 nm. At this time, DOX is also released, which enhances the drug toxicity towards the target cells. In general, the authors successfully established a combined drug delivery platform to improve the therapeutic effect on tumour cells.

##### 5.1.1.2 Lung cancer

Over the past two decades, the use of gene therapy and chemotherapy have become widely popular in the field of molecular medicine. As an anthracycline-based chemotherapy drug, DOX is one of the most widely used anticancer drugs for the treatment of various types of cancer (Koczurkiewicz-Adamczyk et al., 2020; Wei et al., 2020; Yang S et al., 2021). In the research presented by Yuming Yang et al. (Yang Y et al., 2021), a gold nanocage carrier, Au siRNA PAA-AS1411, prepared by highly efficient AS1411 aptamer-modified dsDNA and MMP-2 cleavable peptide, can be loaded with DOX and siRNA to achieve the combination of tumour reactive gene therapy, chemotherapy and photothermal therapy (Figure 3B). In animal experiments, it was confirmed that this combination therapy achieved targeted gene silencing and tumour inhibition in the lung cancer mouse model. This new drug and gene carrier will become the basis for more effective treatment of lung cancer.

##### 5.1.1.3 Gastric cancer

The microenvironment of tumours is extremely different from that of normal tissues, making them difficult to cure. The pH value of tumours is lower than that of normal tissues, which usually hinders the effectiveness of drugs (Koltai, 2020a; Koltai, 2020b). Thus, pH-responsive drugs have received a lot of attention in recent years. In a study (Ding et al., 2020) on gastric adenocarcinoma, Morin was encapsulated with pH sensitive liposomes and Au-aptamer was modified on the surface of the liposomes (Apt-Au@MorinpH-sensitiveliposomes, Apt-Au@MSL). Apt-Au@MSL shows excellent monodispersity and tumour targeting ability (Figure 3C). It can induce apoptosis by triggering the excessive production of ROS and regulating multi-signal crosstalk. Furthermore, it can be released in certain acidic environments through dialysis. The MTT method and nude mouse tumourigenesis experiments showed that SGC-7901 could significantly inhibit the proliferation of SGC-7901. Blood biochemical tests and H&E staining showed that Apt-Au@MSL exhibited no acute toxicity and displayed excellent biocompatibility. AptAu@MSL can be used as a targeted anti-cancer material for future clinical cancer treatment.

#### 5.1.2 Cellular prion protein (PrPC) aptamer conjugated-gold nanoparticles

PrPC is a glycosyl-phosphatidylinositol-anchored cell surface protein, which is related to a variety of cell functions (Mouillet-Richard et al., 2021). The PrPC gene is highly expressed in a variety of cancers, including colorectal cancer (CRC), according to recent studies (Yun et al., 2021). In addition, it was reported that PrPC promotes cancer progression by enhancing cancer cell metastasis, proliferation, and resistance to chemotherapy (Liang et al., 2007; Li et al., 2009; Go and Lee, 2020). These results indicate that PrPC is a promising target for cancer therapy. The anticancer drug DOX is commonly used to treat CRC. However, due to its low response rate and adverse reactions, it is necessary to develop an effective drug delivery system. In one study (Go et al., 2021), PrPC aptamer (Apt)-conjugated AuNPs were synthesized for targeted delivery of DOX to CRC. Mercaptan-terminated PrPC Apt was coupled with AuNPs, and its complementary DNA was then hybridized for drug loading (Figure 3D). Finally, DOX was loaded onto AuNPs to synthesize PrPC Apt-functionalized DOX oligomer AuNPs (PrPC-Apt-DOA). PrPC-Apt-DOA is a spherical NP with an average diameter of 20 nm, which can effectively deliver DOX to CRC cells. To a greater extent, it reduces the proliferation of CRC cells and increases apoptosis, which may replace 5-FU, oxaliplatin, and other anti-cancer drugs.

#### 5.1.3 AuNP–hexahistidine (His)/Glutathione S-transferase (GST)-Apt

Discoidin domain receptor 2 (DDR2), i.e., a collagen-induced receptor tyrosine kinase, has recently been identified as a novel therapeutic target for lung cancer (Kothiwale et al., 2015). The peptide containing the DDR2 functional domain inhibits the proliferation and invasion of cancer cells mediated by DDR2 activation (Valiathan et al., 2012; Borza and Pozzi, 2014). Similarly, overexpression of the transmembrane 2 (JM2) domain blocks the activation of DDR2, thereby reducing the proliferation and invasion of cancer cells *in vitro*. However, it is inefficient to deliver the peptide containing the JM2 domain to cancer cells, which makes it difficult to use the peptide in cancer treatment (Kim et al., 2014). Numerous methods have been developed to transport peptides to mammalian cells (Sikder et al., 2019). A recent study (Kim et al., 2015) reported that peptides containing the DDR2 functional domain or JM2 domain can be effectively delivered to cancer cells through a system based on AuNP-DNA aptamer conjugates.

##### 5.1.3.1 Cervical cancer

Proteins play a central role in maintaining life, and the production of proteins with expression or functional disorder leads to diseased states (Gu et al., 2011; Singh et al., 2019). In a recent study (Ryou et al., 2014) of targeted delivery of protein to restore biological function for the treatment of cervical cancer, a purified His-labelled protein was loaded onto AuNPs conjugated with His-labelled aptamers (AuNP-His Apt) through simple mixing and incubation (Figure 3E). It allows any recombinant protein to be loaded and delivered to the mammalian living system without additional modification. The AuNP-Apt system is also effective for local and systemic targeted delivery of proteins in the body. Local injection of AuNP-Apt loaded with apoptosis inducing BIM protein can effectively inhibit the growth of cervical xenograft tumour derived from HeLa cells. In addition, intravenous injection of AuNP-Apt loaded with epidermal growth factor (EGF) and BIM led to targeted delivery of BIM to xenografts derived from cancer cells overexpressing EGF receptors, with no detectable systemic toxicity. Our research results show that this system can be used as an innovative platform for developing protein based biomedical applications.

##### 5.1.3.2 Lung cancer

In the research study by DaehwanKim et al., a AuNP DNA Apt composite material was developed by coupling an anti-His tag aptamer or an anti-GST aptamer with citrate-stabilized AuNPs (15 nm in diameter) as a universal carrier for the delivery of recombinant proteins *in vivo* (Kim et al., 2015; Ryou et al., 2014). In this study (Kim et al., 2015), the AuNP-Apt-based protein delivery system was used to transport peptides containing DDR2 hexahistidine (His) or glutathione S-transferase (GST) labelled transmembrane proximal 1/2 (TM-JM1/2) domain to lung cancer cell lines (Figure 3F). Through this AuNP-Apt-based system, intracellular delivery of TM-JM1/2 peptide could effectively inhibit collagen-induced DDR2 activation and reduce the proliferation and invasion of cancer cells. Therefore, TM-JM1/2 peptide loaded on the AuNP-Apt conjugate can be a useful strategy for DDR2 positive cancer treatment.

### 5.2 Photothermal therapy

Photothermal therapy is a non-invasive therapy following traditional radiotherapy, chemotherapy, and surgery (Li et al., 2020; Zhi et al., 2020). Temperature is related to DNA damage. When the amount of DNA damage exceeds a certain proportion, it leads to cell death. Tumour cells are more sensitive to cell death caused by thermal stimulation, and it is difficult to dissipate heat to tumour tissues with vascular malformations. Therefore, tumour cells are more likely to die at the same temperature. Photothermal therapy can gather heat in tumour cells through photothermal conversion materials and generate local high heat to accelerate the elimination of tumour cells. Due to its excellent properties, AuNPs can be used as an effective photothermal conversion material for the photothermal treatment of tumours (Table 2) (Dai et al., 2019; Monteiro et al., 2020). The combination of ligand modification can significantly enhance the accumulation of gold nanocomposites in cancer cells and the specificity of targeting tumour cells (Ruttala et al., 2020; Wang et al., 2015; Yang et al., 2015).

#### 5.2.1 Apt-AuNP-GO

For targeted photothermal therapy for human breast cancer (Yang et al., 2015), Apt-AuNP-GO nanocomposites combine the advantages of GO, AuNP, and aptamers. The aptamer can selectively target MUC1 positive human breast cancer cells (MCF-7). In addition, Apt-AuNP-GO has a high photothermal conversion ability for the absorption of NIR light and can exert therapeutic effects on MCF7 cells at ultra-low concentrations without causing adverse effects on healthy cells. Under NIR irradiation, this complex will eventually lead to the reduction of heat shock protein (HSP) in tumour cells, thereby inhibiting the growth of tumour cells. The combination therapy of HSP70 inhibitors can synergistically produce significant anti-tumour effects on breast cancer. This nanocomposite can easily be applied for the construction of Apt-AuNP loaded with HSP70 inhibitors, which can deliver HSP70 inhibitors to tumorigenic regions for chemical therapy. In another study conducted in the same period (Wang et al., 2015), targeted chemotherapy and photothermal therapy were integrated into a multi-functional drug delivery platform. A graphene oxide gold nanoparticle (GO-AuNP) composite modified with DNA aptamers (S2.2aptamer) was successfully synthesized. The GO-AuNP-Apt system loaded with DOX showed thermal stimulation and sustained drug release. *In vitro* cell toxicity tests (A549 and MCF7 cells) showed that combined therapy had the highest tumour cell mortality compared with single photothermal therapy or chemotherapy. In addition, the accumulation of aptamer-modified nanocomposites in cancer cells increased significantly. This study shows that GO-Au nanocomposites modified by aptamers may have the potential for targeted photothermal therapy and chemotherapy for cancer cells.

#### 5.2.2 AS1411 Apt-GNP

AS1411 aptamer-bonded alloy NPs can not only be used as drug carriers, but also play a role in photothermal therapy. Ruttala et al. (2020) created a multi-functional nanoplatform based on a AuNPs assembly. Lonidamine (LND) and GNP coupled with the aptamer AS1411 (AS-LAGN) were used for effective cancer therapy. A number of *in vitro* studies have demonstrated that the chemotherapeutic effect of LND is enhanced by laser irradiation-based hyperthermia. Because of the ability of AuNPs to convert the excited state photon energy into heat energy, the efficacy of photothermal/chemotherapy in animal models can be improved. In addition, immunohistochemical staining analysis confirmed that the ability of AS-LAGN to induce apoptosis/necrosis and ablation in tumour tissues significantly damaged the surrounding healthy tissues. The combination therapy model showed that increased production of ROS resulted in significant apoptosis, higher cytotoxicity, and inhibition of cell migration. In conclusion, the AS-LAGN gold nanoparticles platform may be a promising mitochondrial-based cancer treatment strategy.

## 6 Prospect and challenge of aptamer combined with gold nanoparticles in cancer diagnosis and treatment

The preparation technology of aptamers and AuNPs has gradually matured and has commercialized, and some clinical trials of the application of aptamers and AuNPs in cancer are registered on Clinicaltrial. Clinicaltrials (https://clinicaltrials.gov/) is a global clinical research database funded by the private and public sectors. The FDA requires clinical trial applicants to register on the Clinical trail. A unique identification code is given to each clinical study at the time of registration.

At present, there are eight clinical trials registered on Clinicaltrail on cancer and adapters, including one that has been completed. There are six items related to cancer and AuNPs, and four items have been completed. In clinical trials related to adapters, we have seen the familiar AS1411. In this paper, we found that the adaptor AS1411 plays an important role in cancer diagnosis and research. Similarly, an open randomized controlled phase II study (NCT01034410) on AS1411 combined with cytarabine for the treatment of patients with primary refractory or recurrent acute myeloid leukaemia is also in progress. In the clinical trial on AuNPs, we noticed a clinical study on the safety of NU-0129, a drug based on the spherical nucleic acid (SNA) platform, in patients with recurrent glioblastoma multiforme or gliosarcoma (NCT03020017). The SNA is composed of nucleic acids arranged on the surface of spherical AuNPs. Nucleic acid components can target a gene called Bcl2L129(this gene prevents tumor cells from apoptosis), thus stopping cancer cells from growing. It was further verified that AuNPs can be a good carrier for targeted therapy.

Unfortunately, we did not find any clinical trials on AuNPs and aptamers. However, it is gratifying that in the current immunosensor research on AuNPs aptamers, the detection signal has been significantly enhanced, the response range has been widened, and the detection limit has reduced. After validation in the composite biological matrix, clinical samples were also included for testing. Thus, this method can be used to detect of PSA serum samples in the hospital. These serum samples have been detected by the clinical chemiluminescence method of the hospital and the detection values have been collected. The values of the two methods did not show significant differences, indicating that the aptamer combined with nanocrystal detection system can be used for actual sample analysis (Li M et al., 2021). Thus, a preclinical study of AuNPs combined with aptamers has significant prospects, and there is a possibility of transformation of AuNPs with aptamers to clinical practice.

## 7 Conclusion

The use of nucleic acid aptamers for the treatment of diseases began in 1990. RNA with targeted trans response (TAR) sequences acts as "bait" and can prevent trans-activating proteins from binding to endogenous TAR RNA, thereby inhibiting the expression and replication of HIV genes (Reinemann and Strehlitz, 2014). Since then, the development and application of aptamers has attracted extensive attention. So far, numerous types of aptamers have been screened and applied in various fields of biomedicine. Aptamers can be incorporated into AuNPs to build an applicable platform for molecular imaging, drug delivery, biosensors, and other functions, and to directly be applied for tumour diagnosis and treatment (Kumar et al., 2007).

AuNPs have strong interaction ability with visible light for detecting tumour cells. Under the application of light, free electrons in gold atoms are excited to a collective oscillating state called surface plasmon resonance (SPR), which endow AuNPs with the ability to absorb and scatter visible light. AuNPs combined with aptamers can target tumour cells, and they can be good labels. SERS imaging is performed based on the optical scattering characteristics of AuNPs. In SERS technology, the Raman signals are amplified several times by the ligand AuNP near the molecule (Lee et al., 2009; Kneipp et al., 2010). In imaging diagnosis, the aptamer AuNPs overcomes the shortcomings of short imaging time of contrast agents, nephrotoxicity and lack of targeted molecular imaging. In addition, in comparison with traditional homogeneous catalysts, AuNPs can catalyze various biochemical reactions, promote electron transfer, and shorten the regeneration period. It is most commonly used for colorimetric and electrochemical detection. The size dependent optical properties of AuNPs, such as fluorescence superquenching ability, enhance the performance of optical sensors. The electrochemical response of a biosensor is more sensitive after using AuNPs coating film. The aptamer AuNPs show high specificity, sensitivity, and safety for tumour diagnosis when combined with biosensors. The aptamer AuNPs have shown great promise in the diagnosis and monitoring of tumours.

For the treatment of tumours, researchers have constructed targeted delivery vectors for various chemotherapy drugs, siRNA, and polypeptides using aptamer AuNPs. In terms of cancer chemotherapy, the common problems of chemotherapy drugs include non-specific cytotoxicity to normal cells, low tumour cohesion, and drug resistance (Shapira et al., 2011). Aptamer AuNPs provide a novel way to improve the efficiency of drug therapy. They can deliver effective doses of drugs to target cells, reduce side effects of anti-cancer drugs, improve their therapeutic effects, and avoid or reduce the need for repeated drug administration. In addition, photothermal therapy has become an effective cancer treatment method. In recent years, AuNPs have shown outstanding performance in non-invasive photothermal therapy of tumours due to their unique plasmonic resonance characteristics and good biocompatibility. *In vitro* cytotoxicity test showed that the appropriate ligands of AuNPs could play the role of both drug chemotherapy and photothermal therapy, and the mortality of tumor cells in combination therapy was higher than that in drug chemotherapy or photothermal therapy alone; additionally, the aptamer modification could significantly enhance the accumulation of nanocomposite materials in cancer cells. The GO-Au nanocomposites modified by aptamers may have potential in targeted photothermal therapy and chemotherapy for cancer cells. The radiosensitization potential of AuNPs has been supported and confirmed by various experimental data (Chen et al., 2020). Previous research showed that the biological distribution of AuNPs is one of the most important factors affecting radiation efficiency. However, there is still a lack of research on radiosensitization of AuNPs as aptamers.

In conclusion, in the current state, many preliminary basic experiments have proved the advantages and prospects of aptamer AuNPs in tumour detection and treatment. Although registered clinical trials on aptamer binding AuNPs are still in the starting stage, some basic studies have included clinical samples for verification. It is believed that research on aptamer AuNPs will make more progress, and the research results will be transformed and applied to the laboratory, for imaging diagnosis, and in clinical precision treatment.
