# Supplementary material for: Aptamer-conjugated gold nanoparticles and their diagnostic and therapeutic roles in cancer
Source: Front Bioeng Biotechnol. 2023 Jan 19;11:1118546. doi: 10.3389/fbioe.2023.1118546 (PMC9892635; doi:10.3389/fbioe.2023.1118546)
Supplement: Supplementary file 1 [file DataSheet1.doc]

**Figure S2A**

**
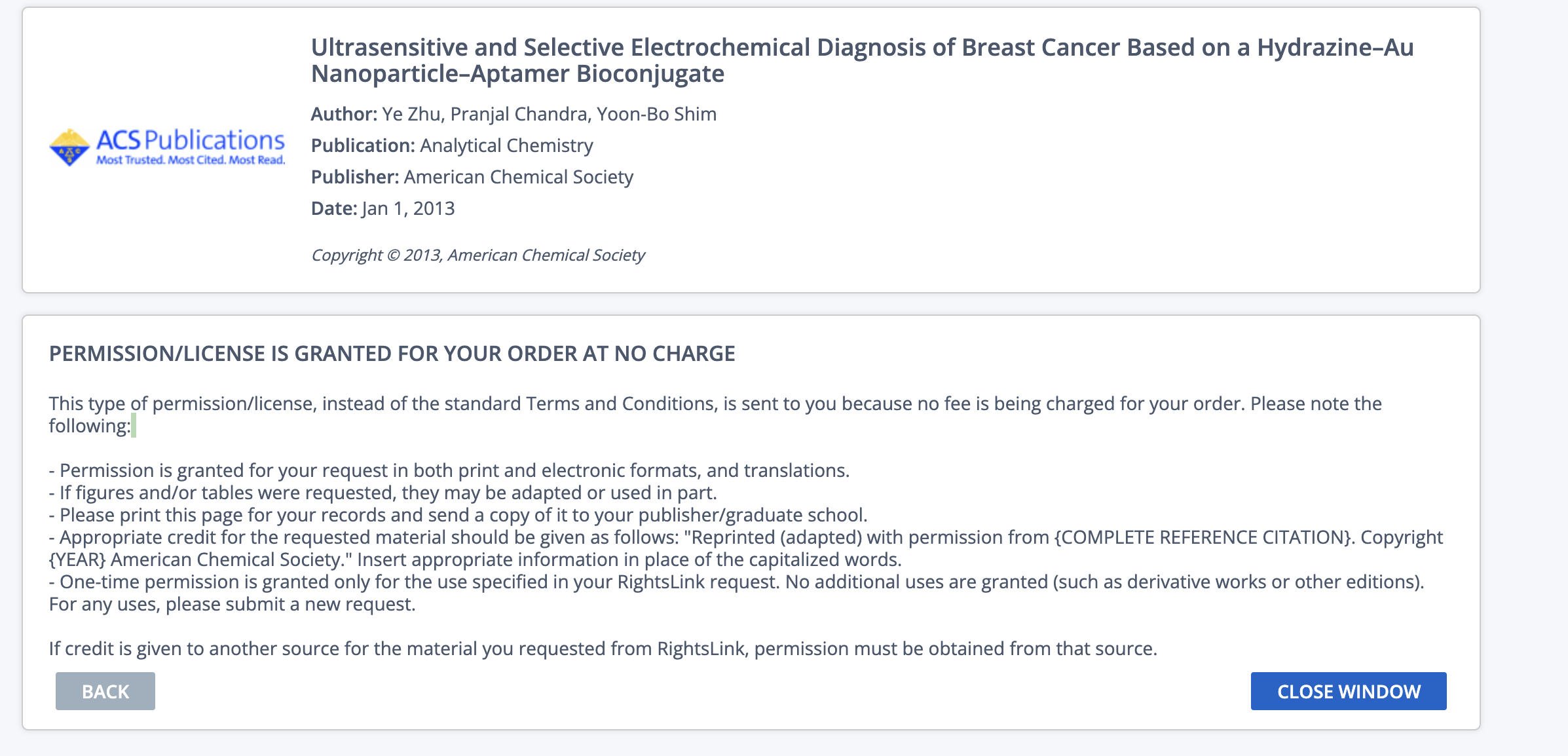
**

**Figure S2B**

**
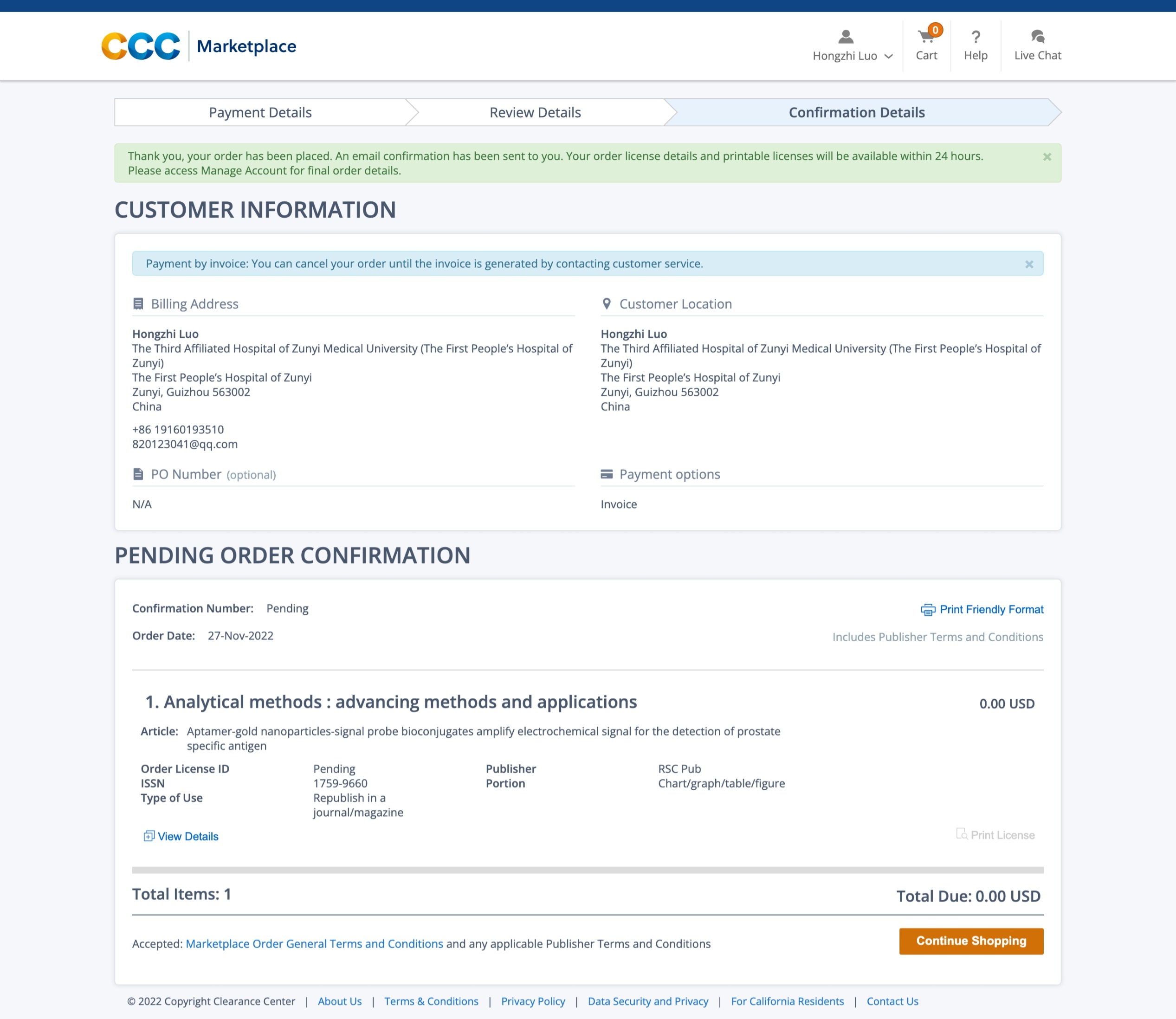
**

**Figure S2C**

**
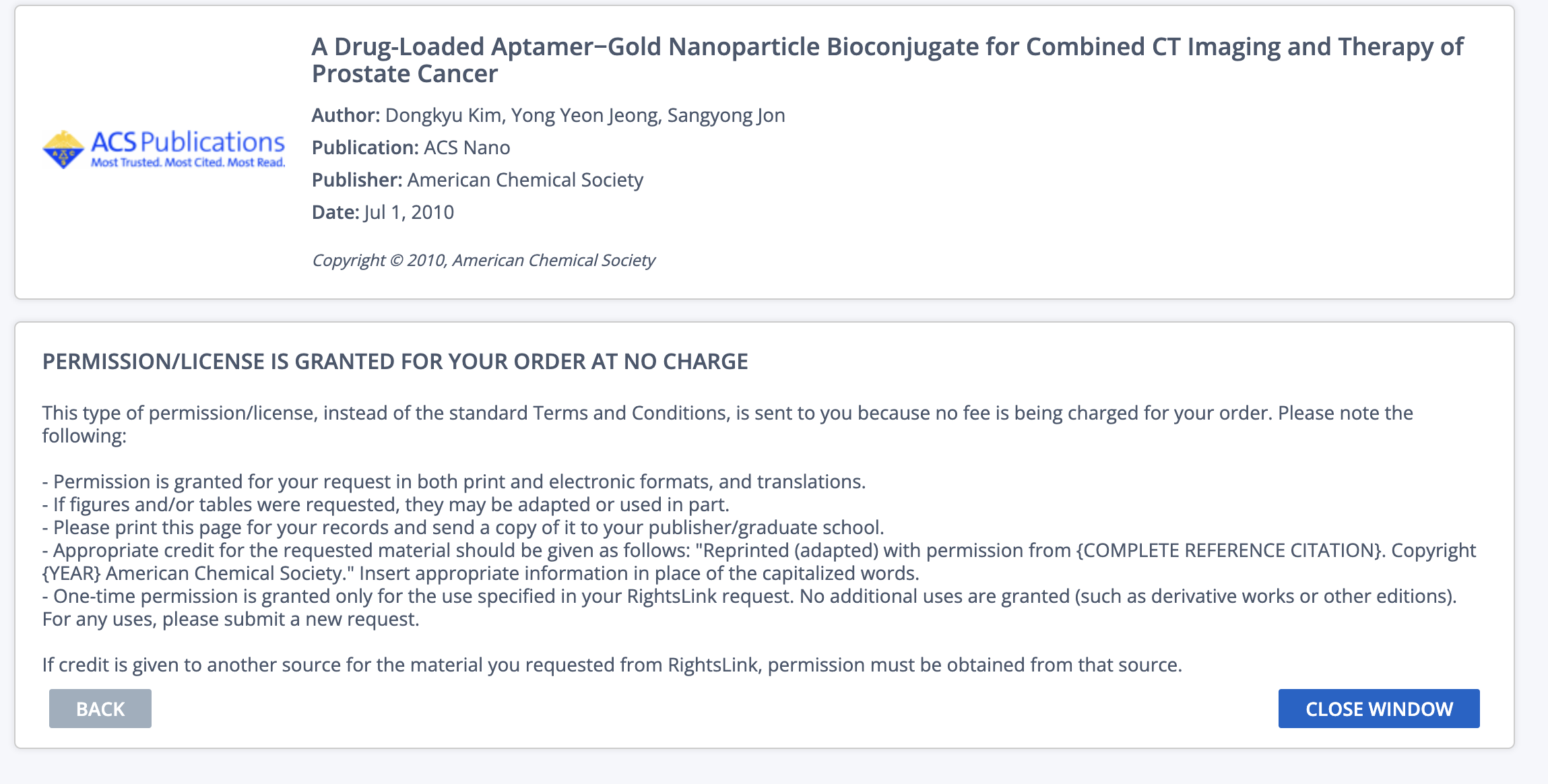
**

**Figure S2D**

**
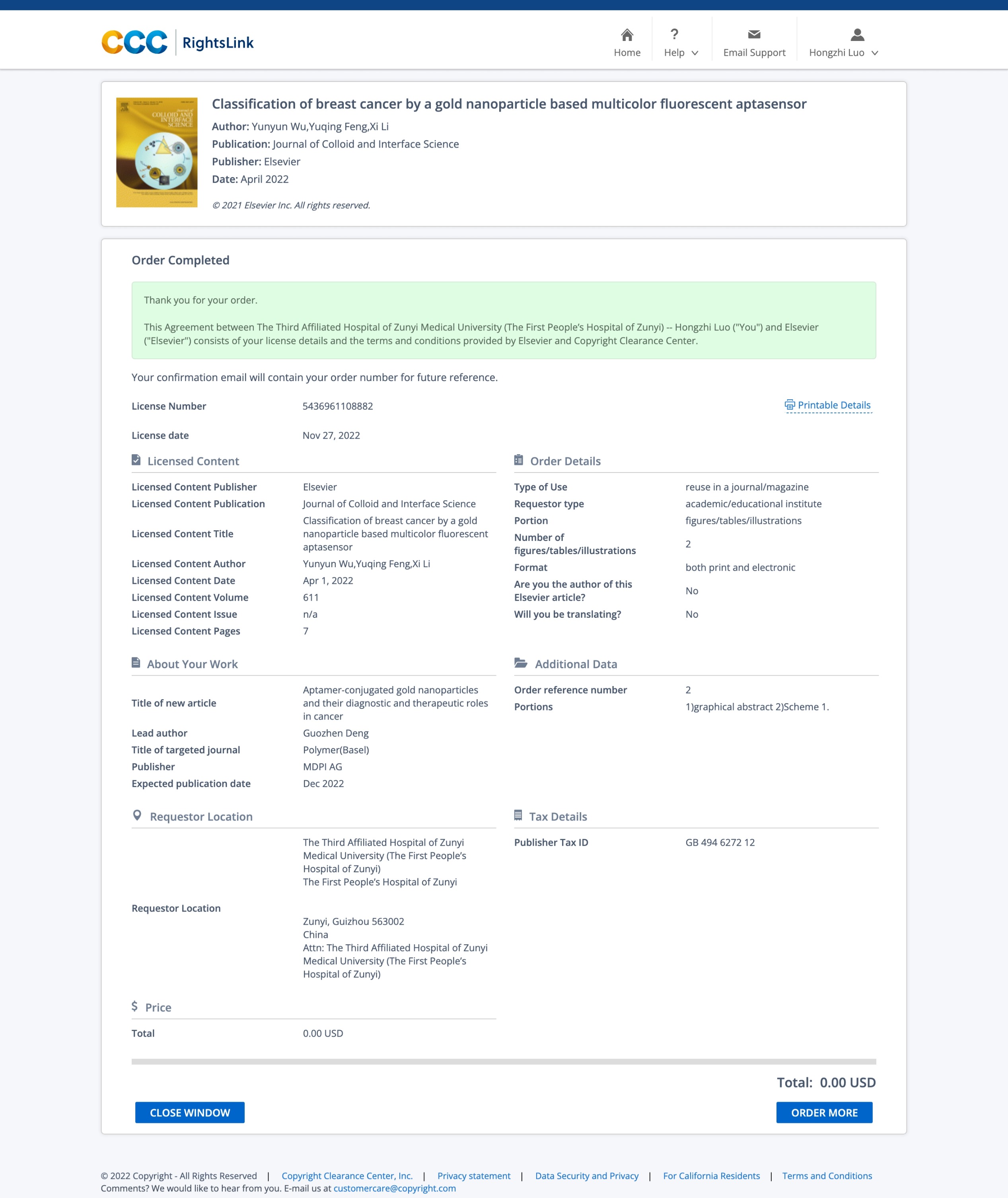
**

**Figure S2E**

**
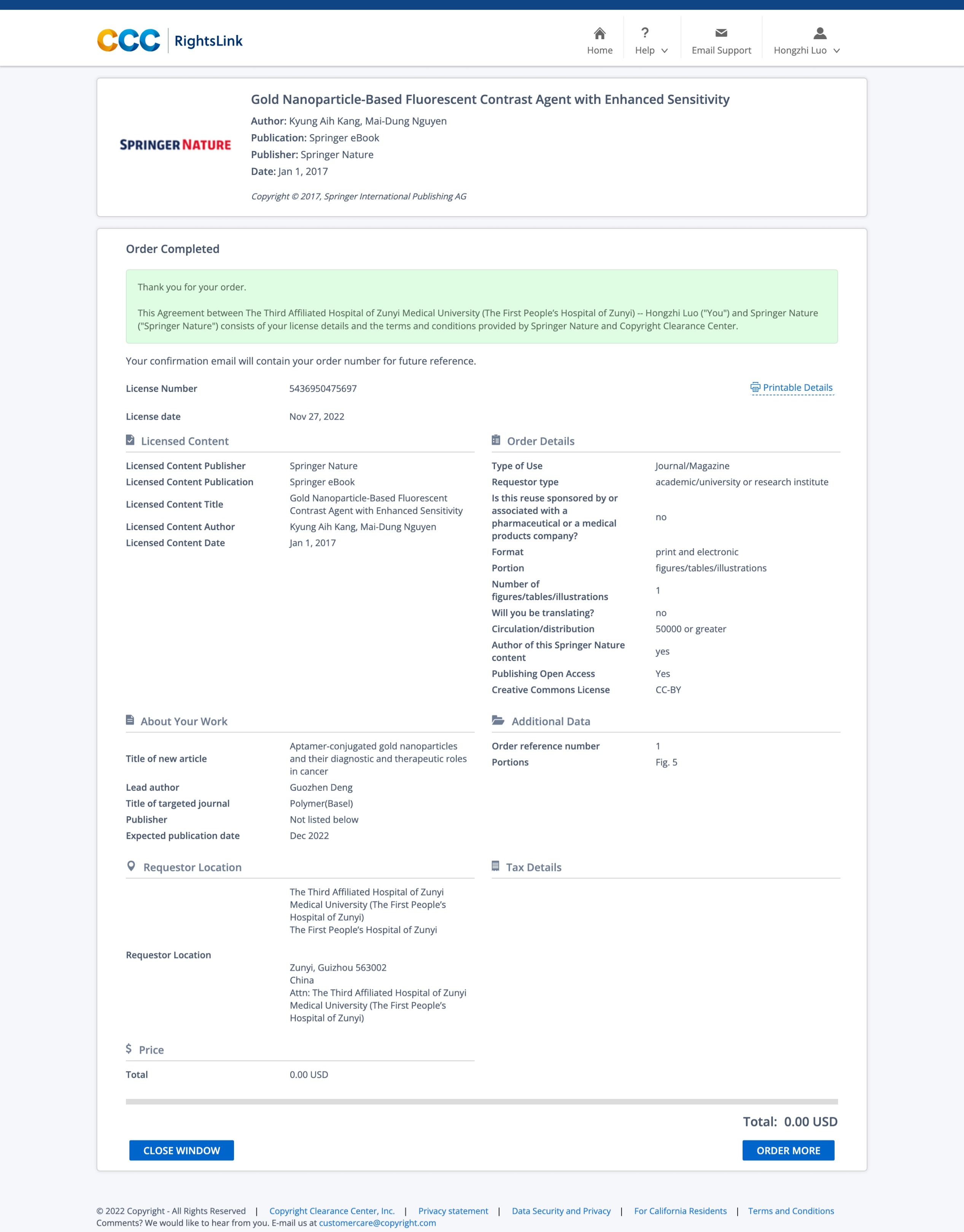
**

**Figure S2F**

**
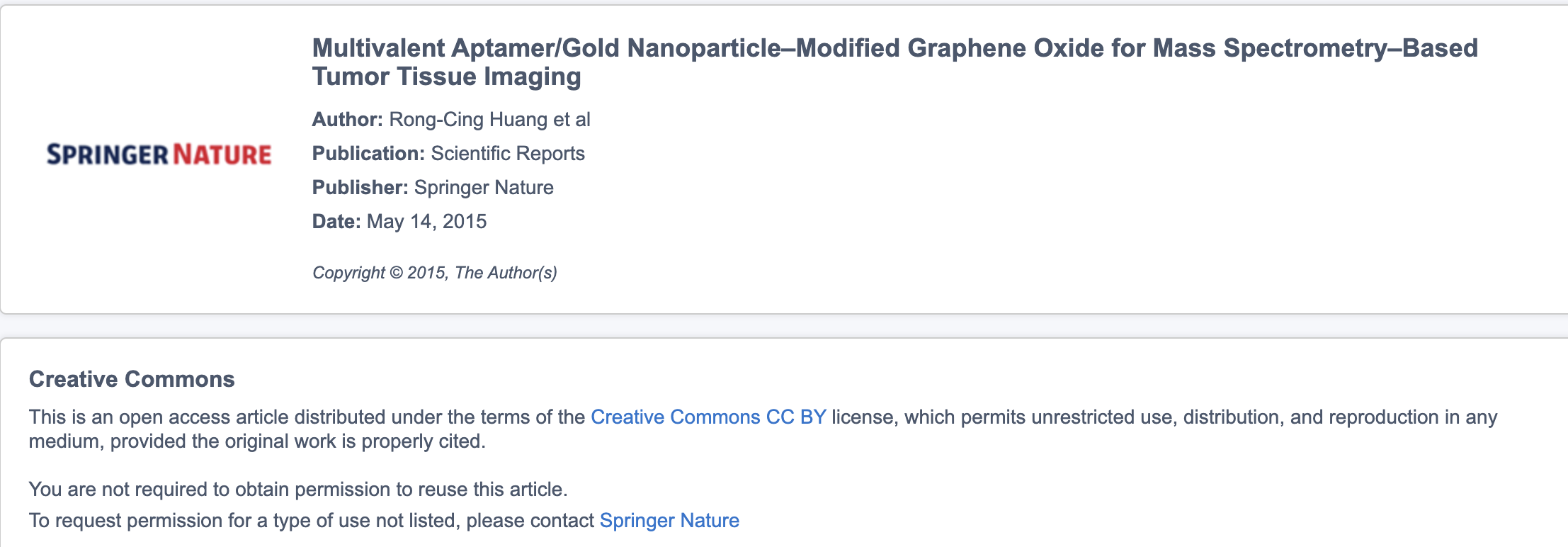
**

**Figure S3A**

**
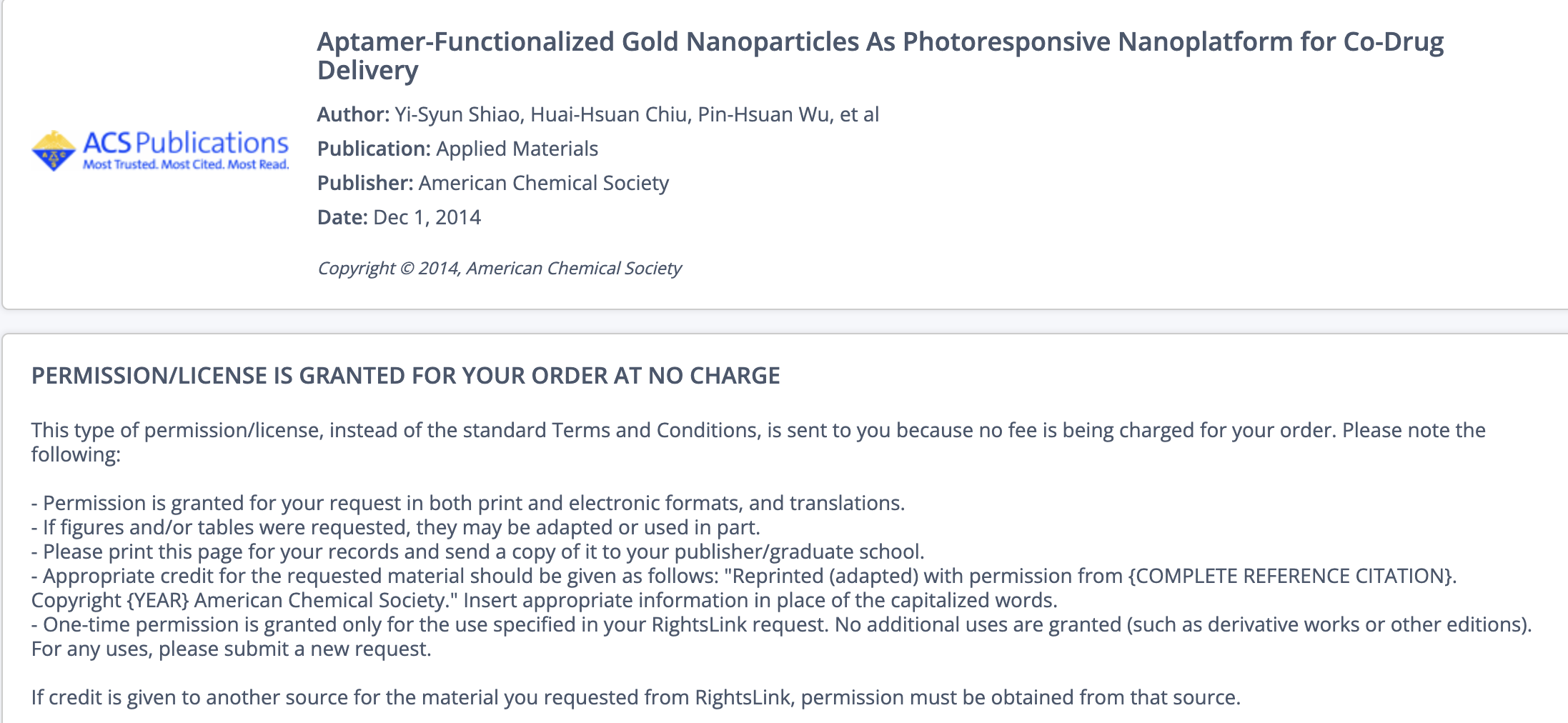
**

**Figure S3B**

**
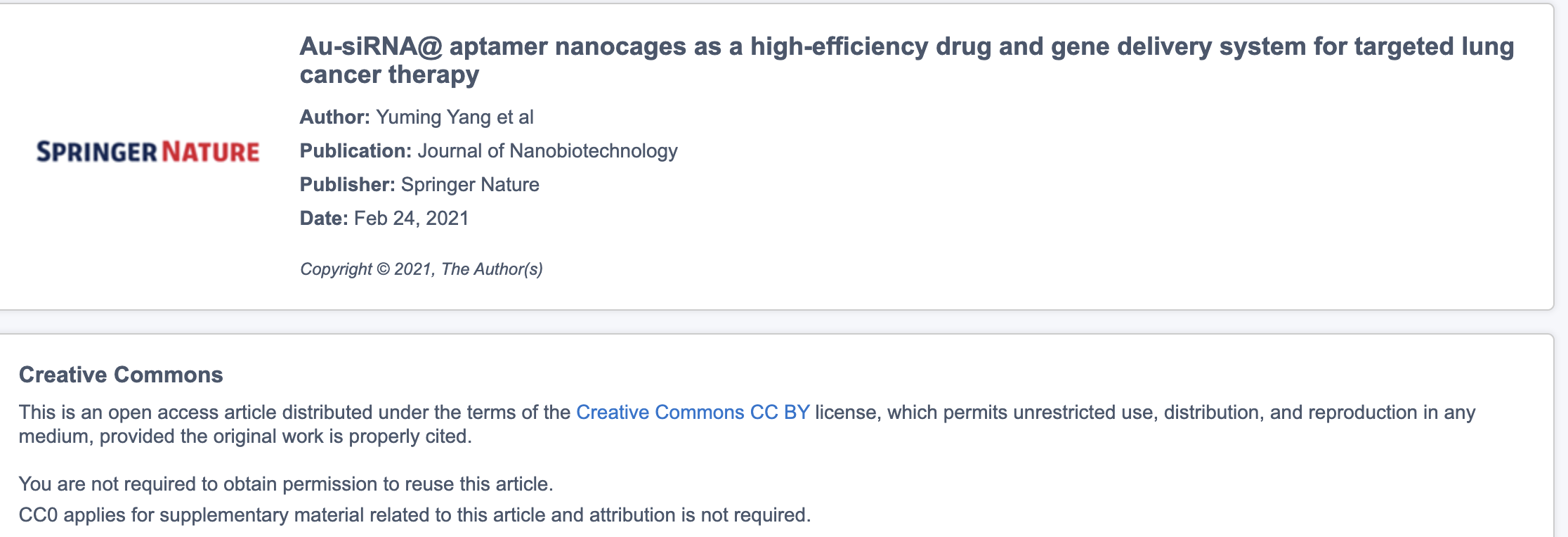
**

**Figure S3C**

**
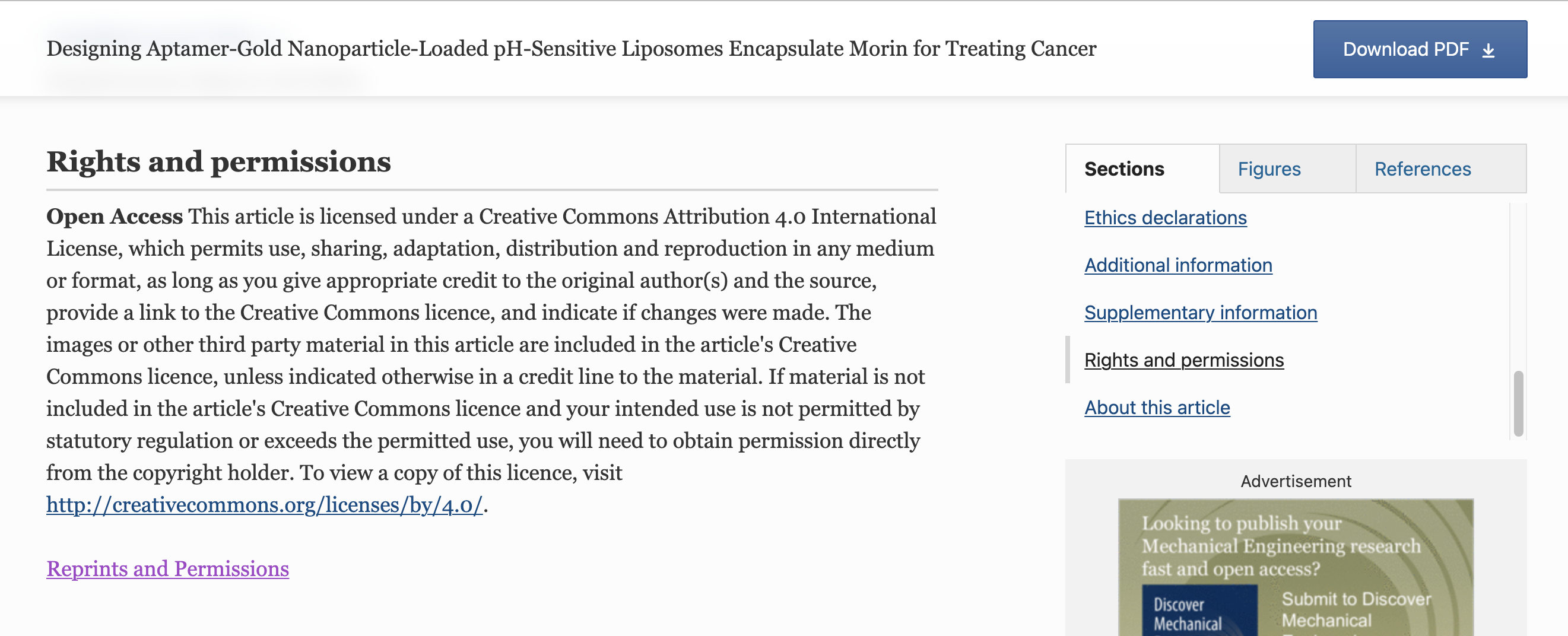
**

**Figure S3D**

**
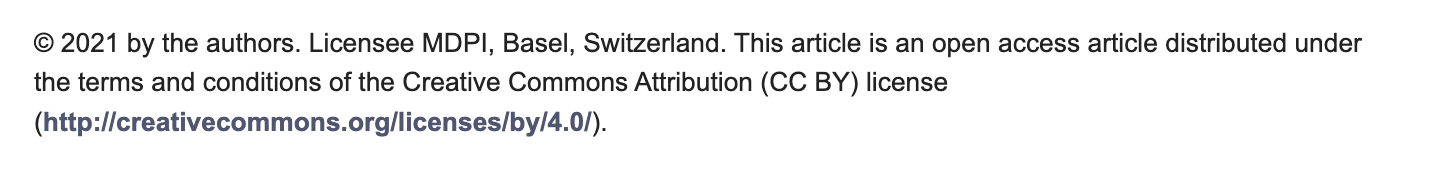
**

**
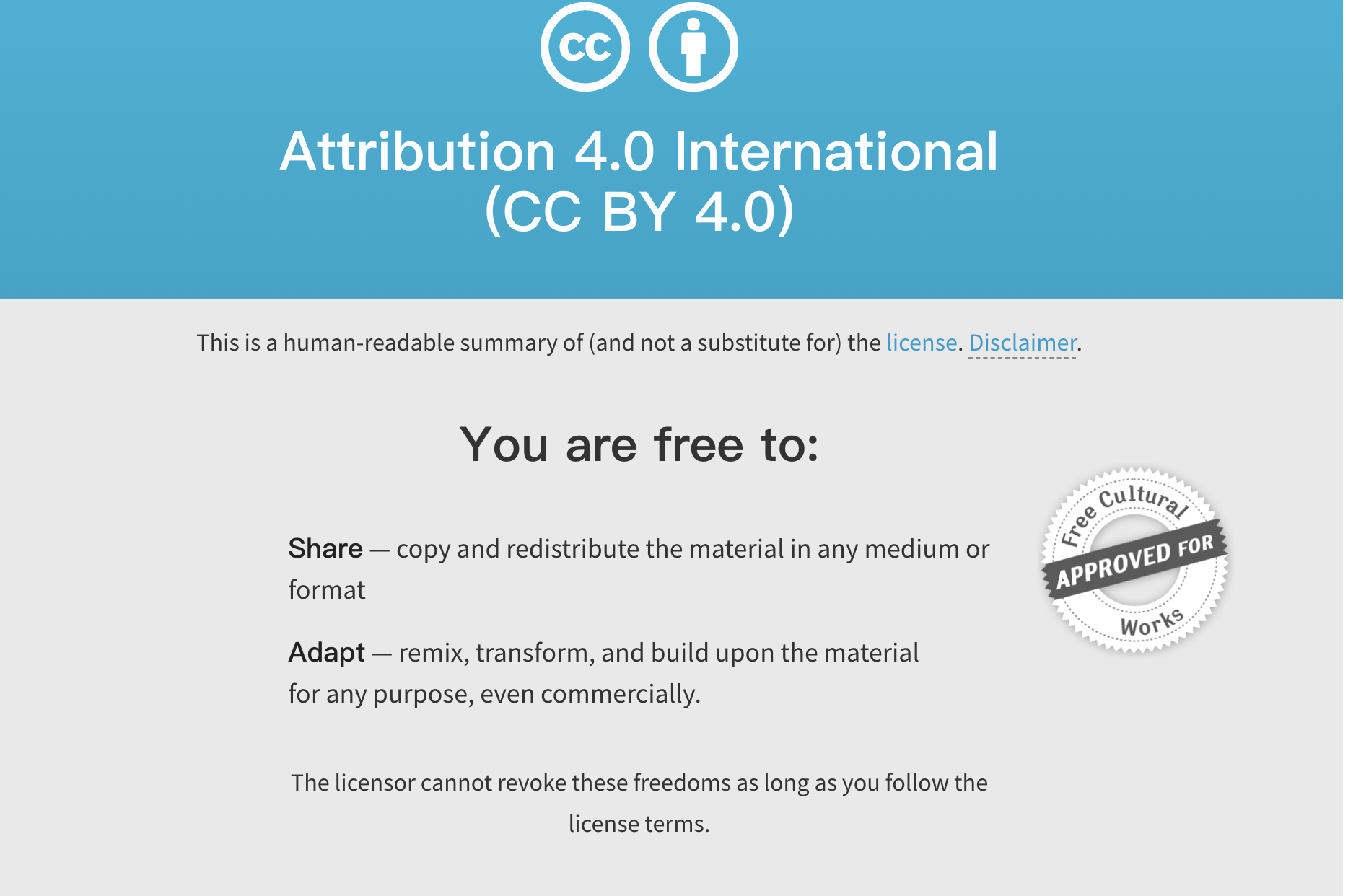
**

**Figure S3E**

**
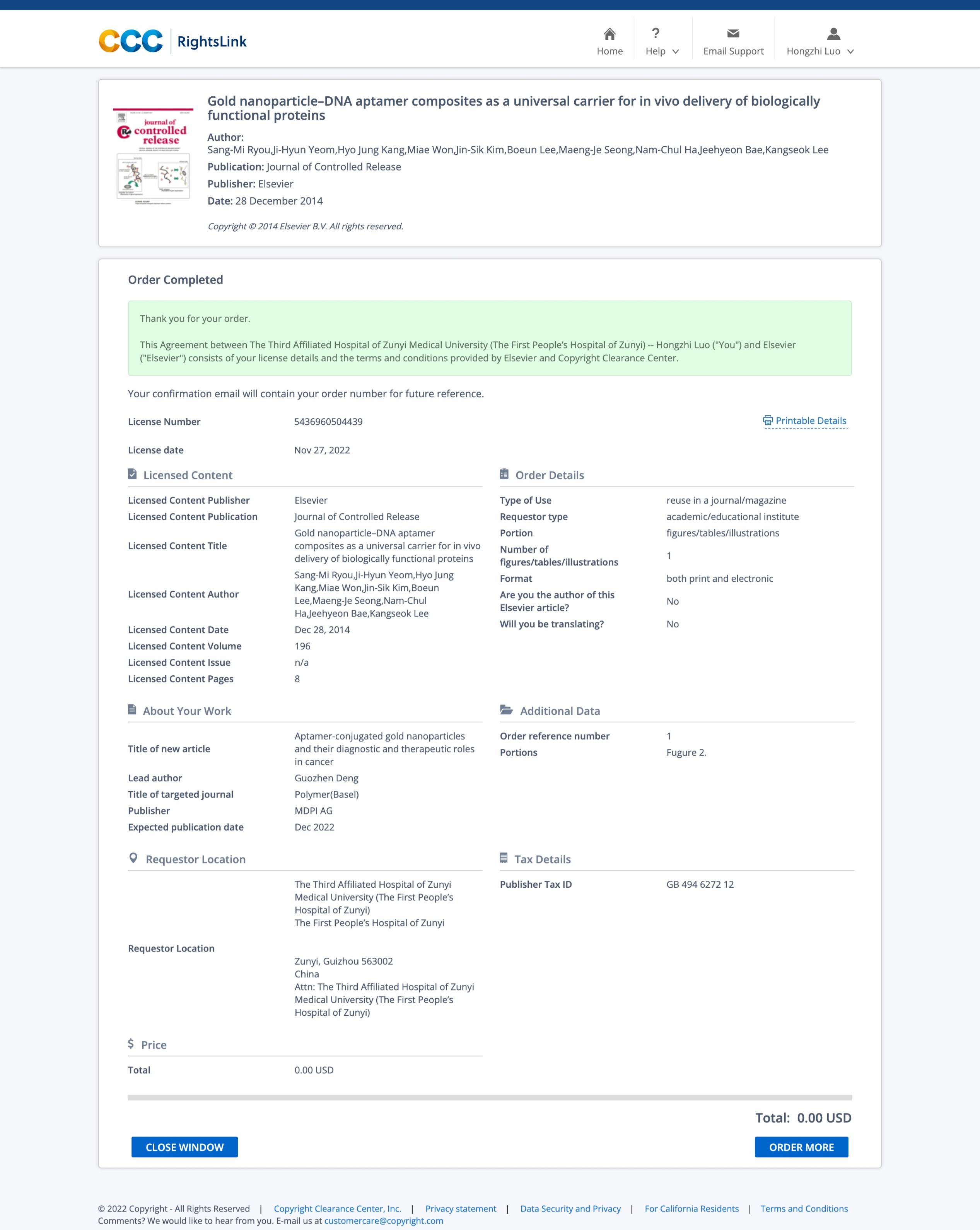
**

**Figure S3F**

**
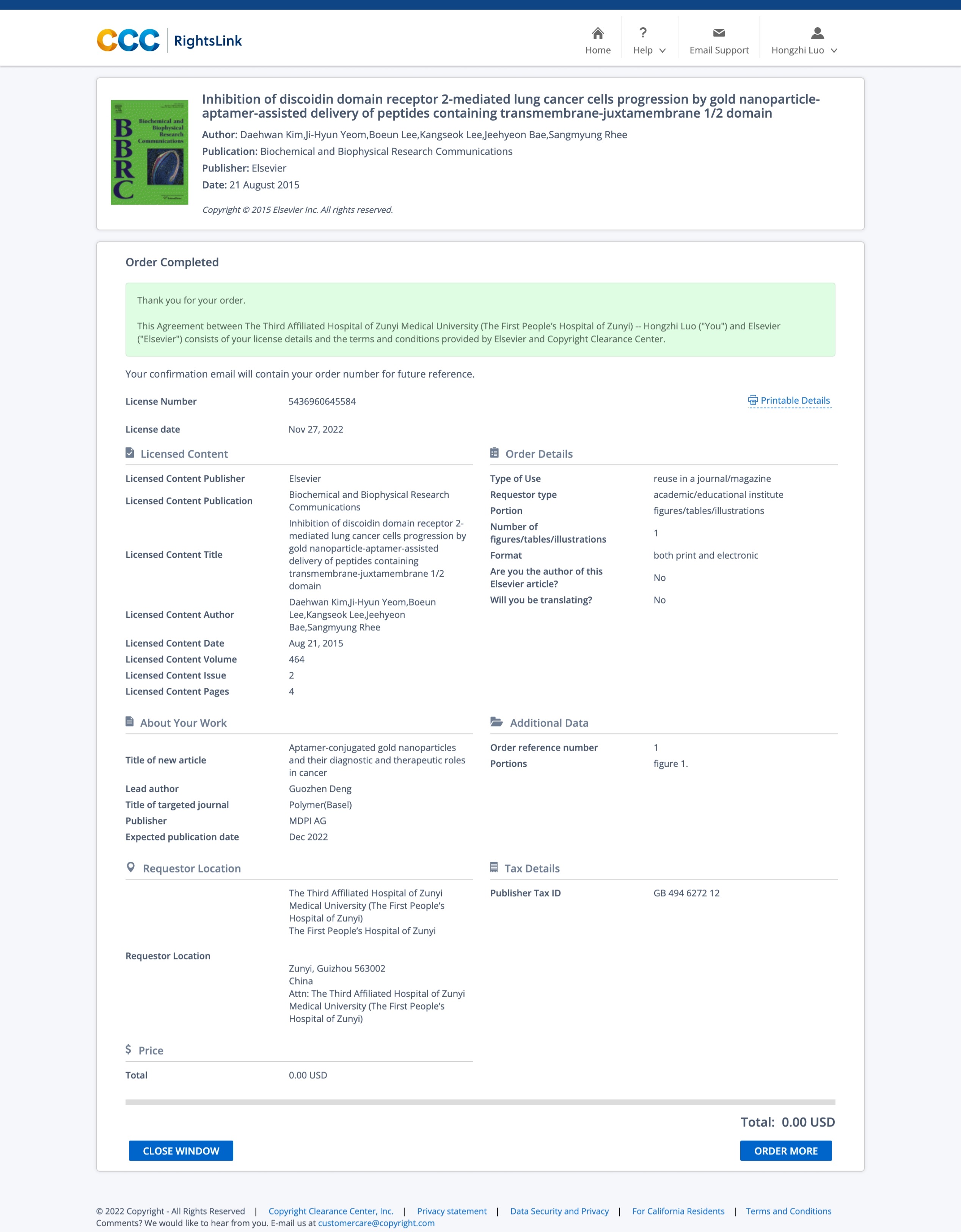
**
